# Supplementary material for: Ectopic Expression of CrPIP2;3, a Plasma Membrane Intrinsic Protein Gene from the Halophyte Canavalia rosea, Enhances Drought and Salt-Alkali Stress Tolerance in Arabidopsis
Source: Int J Mol Sci. 2021 Jan 8;22(2):565. doi: 10.3390/ijms22020565 (PMC7827864; doi:10.3390/ijms22020565)

**Supplementary Material Information**

Table S1. *Cis*-acting elements in *CrPIP2;3*’s promoter region.

Table S2. Sequence information of other plant PIPs used in this study.

Table S3. Primer sequences used in this study.

Figure S1. Sequence analysis of the putative promoter region of *CrPIP2;3*. (A) The distribution of abiotic stress-regulatory *cis*-acting elements, which were identified in the *CrPIP2;3* promoter region (ATG 2000 bp upstream). (B) The *cis*-acting element categories and possible functions in this study.

Figure S2. Differential expression of *CrPIP2;3* various organs (young root, young leaf, leaf, vine, mature leaf, flower, and young legume).

Figure S3. Antioxidant analysis of *CrPIP2;3* transgenic lines and WT under abiotic stresses. (A) Analysis of the expression levels of anti-oxidative stress genes in *CrPIP2;3* transgenic lines (*CrPIP2;3* *OX 3#*, *OX 6#* and *OX 10#*) and WT plants by qRT-PCR under normal and salt/alkali/osmotic conditions. (B-C) Oxidative stress analyses of the *CrPIP2;3* transgenic OX lines (*CrPIP2;3* *OX 3#*, *OX 6#* and *OX 10#*) and WT plants. Histochemical staining assays were used to detect H2O2 and O2− in the leaves by NBT (B) or DAB (C) staining, respectively.

Figure S4. Simple map of *CrPIP2;3*-pYES DEST52 (A) and *CrPIP2;3*-pEGAD (B).

**Table S1**

| **No** | **cis-Element** | **Location (upstream of ATG)** | **Core sequence (5’ to 3’)** | **Putative function (species)** |
| --- | --- | --- | --- | --- |
| 1 | TATA-Box | -276 | TATA | Core promoter element around -30 of transcription (*Arabidopsis thaliana*) |
| 2 | CAAT-Box | -318 | CAAAT | Common cis-acting element in promoter and enhancer regions (*Arabidopsis thaliana*) |
| 3 | ERE | -419, -531, -544, -1611, -1740, -1879 | ATTTTAAA | Ethylene-responsive element (*Nicotiana glutinos*) |
| 4 | CGTCA-motif | -1087 | CGTCA | Cis-acting regulatory element involved in the MeJA-responsiveness (*Hordeum vulgare*) |
| 5 | TGACG-motif | -1087 | TGACG | Cis-acting regulatory element involved in the MeJA-responsiveness (*Hordeum vulgare*) |
| 6 | TCA-element | -84 | CCATCTTTTT | Cis-acting element involved in salicylic acid responsiveness (*Nicotiana tabacum*) |
| 7 | MYB | -1842 | TAACCA | MYB binding site involved in drought-inducibility (Arabidopsis thaliana) |
| 8 | MYC | -125, -1145, -1229, -1626, -2076, -2087 | CATTTG | MYC binding site involved in drought-inducibility (*Arabidopsis thaliana*) |
| 9 | CCAAT-Box | -662 | CAACGG | MYBHv1 binding site (*Hordeum vulgare*) |
| 10 | ARE | -1128, -2062 | AAACCA | Cis-acting regulatory element essential for the anaerobic induction (*Zea mays*) |
| 11 | Box III | -1048 | atCATTTTCACt | Protein binding site (*Pisum sativum*) |
| 12 | CAT-box | -932 | GCCACT | Cis-acting regulatory element related to meristem expression (*Arabidopsis thaliana*) |
| 13 | Box 4 | -138, -578, -1009, -1043, -1198, -1827, -1932, -1196, -2116 | ATTAAT | Part of a conserved DNA module involved in light responsiveness (*Petroselinum crispum*) |
| 14 | Sp1 | -217 | GGGCGG | Light responsive element (*Oryza sativa*) |
| 15 | GATA-motif | -1044, -1166 | GATAGGA | Part of a light responsive element  (*Arabidopsis thaliana*) |
| 16 | I-box | -1164, -1165 | ccttatcct | Part of a light responsive element  (*Arabidopsis thaliana*) |
| 17 | TCCC-motif | -588 | TCTCCCT | Part of a light responsive element  (*Spinacia oleracea*) |

**Table S2**

**Table S3**

| **Primer ID** | **Sequence (from 5′ to 3′)** | **Purpose** |
| --- | --- | --- |
| CrPIP2;3RTF | AGGGGTTAACCTTGTGAGCG | Primer pair for qRT-PCR of *CrPIP2;3* in *C. rosea* |
| CrPIP2;3RTR | TGGTCATCCCAGGCTTTGTC |  |
| CrEF-αRTF | GACCTTCTTCGTTTCTCGCA | Primer pair for qRT-PCR of reference gene CrEF-α in *C. rosea* |
| CrEF-αRTR | CGAACCTCTCAATCACACGC |  |
| AtACT2RTF | GGTAACATTGTGCTCAGTGGTGG | Primer pair for qRT-PCR of reference gene AtActin2 (At3g18780) in Arabidopsis |
| AtACT2RTF | AACGACCTTAATCTTCATGCTGC |  |
| CrPIP2;3OXF | GGCAGCGGCCGAATTCATGGCTAAAGACGTTGAGCAGG | Primer pair for cloning the full-length CDS of *CrPIP2;3* and construction of *CrPIP2;3*-pEGAD, *Eco*RI and *Bam*HI sites were underlined |
| CrPIP2;3OXR | CAGTTATCTAGGATCCTCATGAAGCGTTGCTCCTGAAGG |  |
| HAI2RTF | ACGGGCTATGGGACGTAGTG | Primer pair for qRT-PCR of *HAI2* (*At1g07430)* in Arabidopsis |
| HAI2RTR | ACACATGCGCACCATCGTA |  |
| RD26RTF | AGTTCGATCCTTGGGATTTG | Primer pair for qRT-PCR of *RD26* ( *At4g27410*) in Arabidopsis |
| RD26RTR | ACCCGTTGCTTTCCAATAAC |  |
| RD29BRTF | AAGGAGACGCAACAAGGG | Primer pair for qRT-PCR of *RD29B* (*At5g52300*) in Arabidopsis |
| RD29BRTR | ACGGTGGTGCCAAGTGAT |  |
| ANAC19RTF | CAACTGTGGCTACCTGAAGACGG | Primer pair for qRT-PCR of *ANAC19* (*At1g52890*) in Arabidopsis |
| ANAC19RTR | CAAACGAGTCAACACCATAACCCT |  |
| CAT1RTF | CGCCATGCCGAAAAATACCC | Primer pair for qRT-PCR of *CAT1* (*At1g20630*) in Arabidopsis |
| CAT1RTR | CTTGCCTGTCTGAATCCCAGGAC |  |
| CSD1RTF | TGATGGAACTGCCACCTTCACA | Primer pair for qRT-PCR of *CSD1* (*At1g08830*) in Arabidopsis |
| CSD1RTR | ATGGCCTCCCTTTCCGAGGT |  |
| APX1RTF | GGACGATGCCACAAGGAT | Primer pair for qRT-PCR of *APX1* (*At1g07890*) in Arabidopsis |
| APX1RTR | CGACCAAAGGACGGAAAA |  |
| FSD1RTF | GCTCGGCTCTTTCCCATTGC | Primer pair for qRT-PCR of *FSD2* (*At4g25100*) in Arabidopsis |
| FSD1RTR | CAGCTTCCCAAGACACAAGATTGG |  |

**Figs S1**


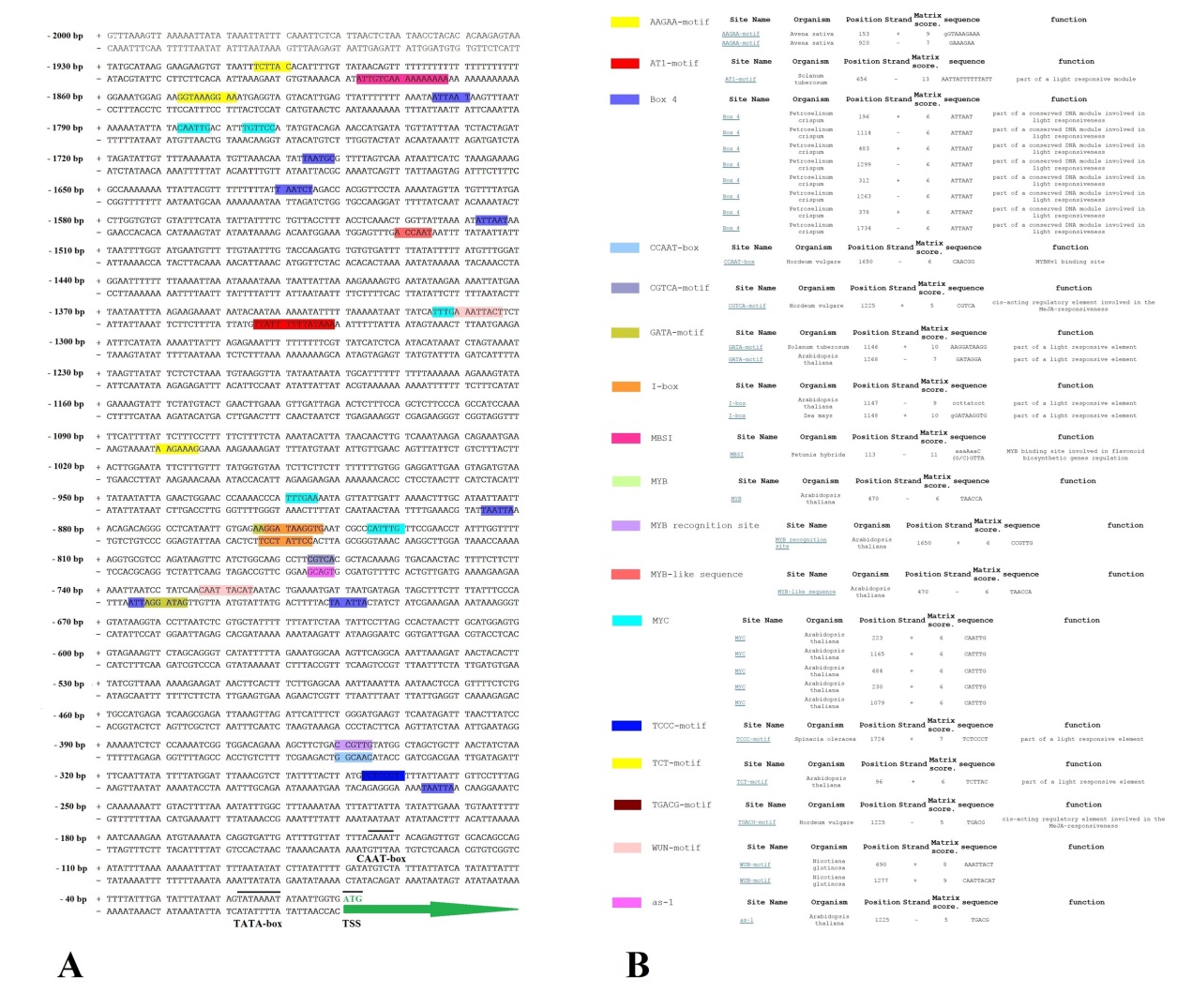


**Figs S2**


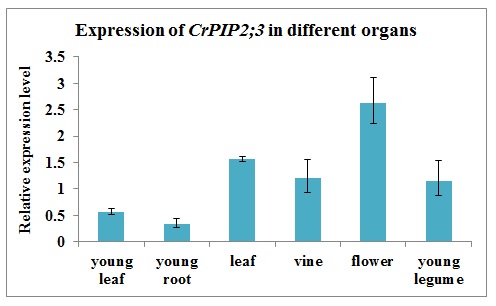


**Fig. S3**


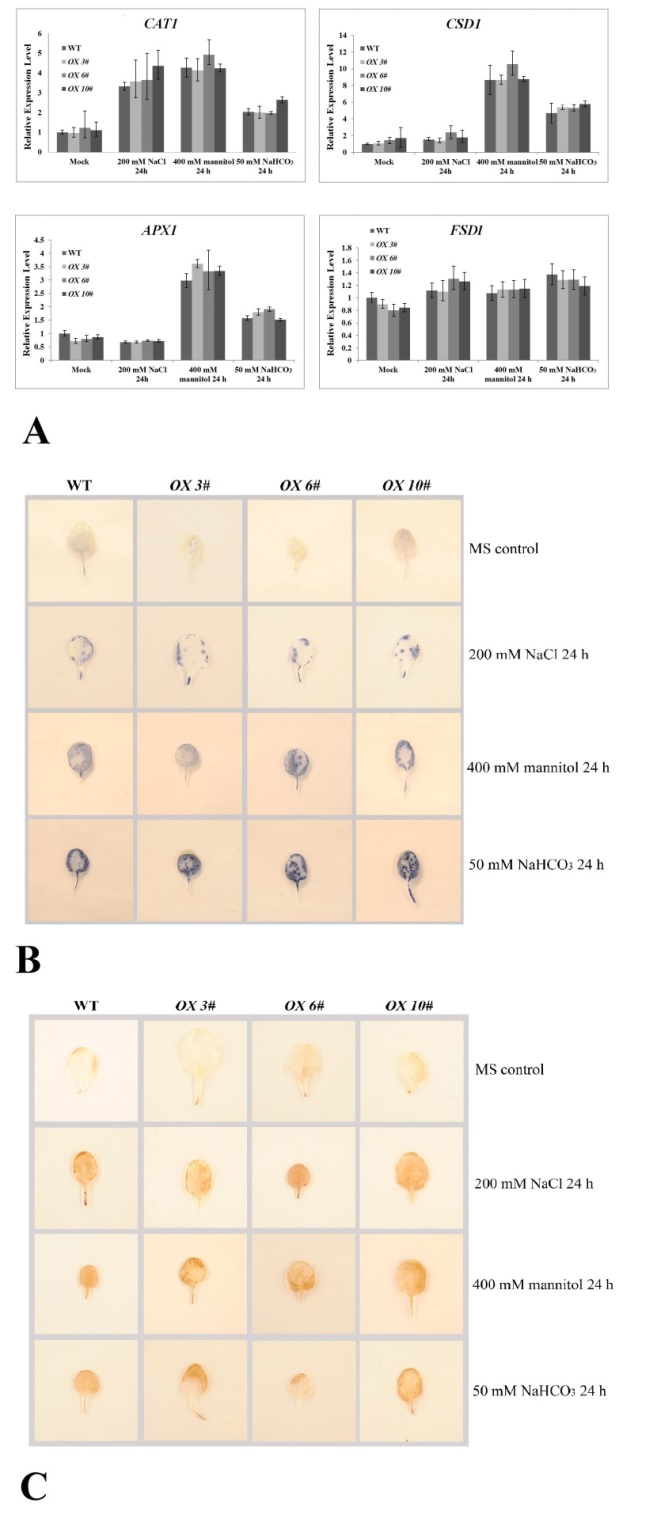


**Fig. S4**


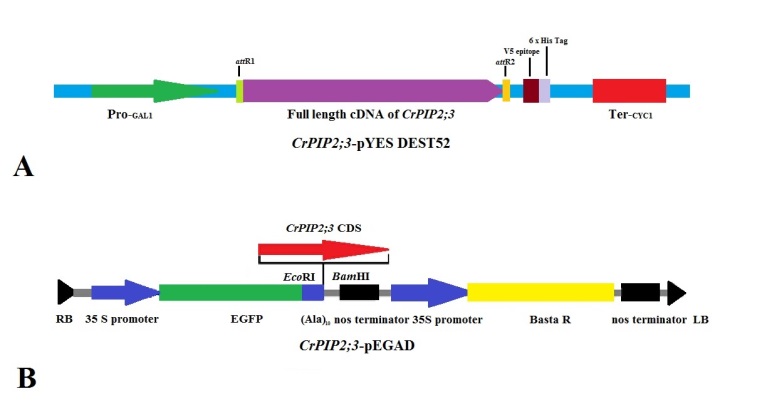

Supplement: Supplementary file 1 [file ijms-22-00565-s001.zip › Supplementary materials of ijms-1049840---R1 without tracked revisions.docx]
